# Cloning, overexpression and biocatalytic exploration of a novel Baeyer-Villiger monooxygenase from *Aspergillus fumigatus* Af293

**DOI:** 10.1186/2191-0855-3-33

**Published:** 2013-06-14

**Authors:** Maria Laura Mascotti, Maximiliano Juri Ayub, Hanna Dudek, Marcela Kurina Sanz, Marco W Fraaije

**Affiliations:** 1 INTEQUI-CONICET, Facultad de Química Bioquímica y Farmacia, Universidad Nacional de San Luis, CP 5700 San Luis, Argentina; 2 IMIBIO-CONICET, Facultad de Química Bioquímica y Farmacia, Universidad Nacional de San Luis, CP 5700 San Luis, Argentina; 3 Laboratory of Biochemistry, Groningen Biomolecular Sciences and Biotechnology Institute, University of Groningen, Nijenborgh 4, 9747AG Groningen, The Netherlands

**Keywords:** Eukaryotic BVMO, *Aspergillus*, Baeyer-Villiger oxidation, Kinetic resolution, Sulfide oxidation

## Abstract

The presence of several putative Baeyer-Villiger Monooxygenases (BVMOs) encoding genes in *Aspergillus fumigatus* Af293 was demonstrated for the first time. One of the identified BVMO-encoding genes was cloned and successfully overexpressed fused to the cofactor regenerating enzyme phosphite dehydrogenase (PTDH). The enzyme named BVMO_Af1_ was extensively characterized in terms of its substrate scope and essential kinetic features. It showed high chemo-, regio- and stereoselectivity not only in the oxidation of asymmetric sulfides, (*S*)-sulfoxides were obtained with 99% *ee*, but also in the kinetic resolution of bicyclo[3.2.0]hept-2-en-6-one. This kinetic resolution process led to the production of (1*S*,5*R*) normal lactone and (1*R*,5*S*) abnormal lactone with a regioisomeric ratio of 1:1 and 99% *ee* each. Besides, different reaction conditions, such as pH, temperature and the presence of organic solvents, have been tested, revealing that BVMO_Af1_ is a relatively robust biocatalyst.

## Introduction

Baeyer-Villiger monooxygenases (BVMOs) are flavin-dependent enzymes capable of catalyzing the insertion of an oxygen atom between a C-C bond in carbonyl compounds (Baeyer-Villiger oxidation) such as ketones and aldehydes, yielding esters and lactones (Kamerbeek 2003a). These enzymes can also oxidize C = C bonds to epoxides (Colonna 2002) and heteroatom-containing molecules; e.g. organic sulfides, amines and boron compounds (Brondani 2011). Therefore, BVMOs are able to work on either electron-poor or electron-rich molecules. Remarkably, different mechanisms have been proposed for these O_2_-driven reactions (Ottolina 2005). In the first case, a nucleophilic attack takes place resembling the chemical Baeyer-Villiger reaction conducted by peracids. Unlike this, to oxidize a heteroatom, an electrophilic attack might take place. A common feature among these mechanisms is the strict dependence of a reduced nicotinamide coenzyme (NAD(P)H) for the reduction of the flavin cofactor (FAD). In a subsequent step, the reduced flavin reacts rapidly with molecular oxygen to form a reactive peroxyflavin intermediate. Only after the oxygenation of the substrate, the enzyme reverts to the oxidized state. Subsequently the oxidized NAD(P)^+^ coenzyme is released. This latter process has been shown to be rate-determining in the kinetic mechanism studied for the BVMOs, cyclohexanone monooxygenase (CHMO) (Sheng 2001) and phenylacetone monooxygenase (PAMO) (Torres Pazmiño et al. [Bibr B34][Bibr B35]).

BVMOs are an attractive alternative in organic synthesis due to their relaxed substrate acceptance, their high chemo-, regio- and enantioselectivity, as well as their optimal activity in mild and environmentally-friendly conditions. Because of this, the last few years have witnessed a steady growth in research efforts towards discovery and engineering of BVMOs (Leisch 2012Balke 2012). By random mutagenesis and site directed evolution, it has been possible to expand the substrate specificity, as well as to improve and change the stereoselectivity of cyclopentanone monooxygenase (CPMO) (Clouthier and Kayser 2006) and CHMO (Zhang 2013), among others. Moreover, the designing of chimeric BVMOs by using the scaffold of the thermostable PAMO and introducing novel selectivities of other BVMOs has been developed. Interestingly, it has been demonstrated that the properties of chimeric BVMOs are not directly predictable from the parental enzyme features (van Beek 2012). Concerning the finding of novel BVMOs, the most striking, recently reported example is the large set of rhodococcal BVMOs that has been produced and characterized (Szolkowy 2009; Riebel 2012). The genome of *Rhodococcus jostii* was found to contain 22 type I BVMOs which were all functionally expressed and their biocatalytic properties assessed. This example has revealed that the sequence diversity among this class of flavoprotein monooxygenases is extremely broad, even within a single genome. Despite the recent efforts in discovery and engineering of novel enzymes, the number of recombinant available BVMOs is still rather modest and these are mainly derived from bacterial species.

Based on the exploration of sequenced genomes, it was proposed that fungal ones are rich in BVMO-encoding genes, especially within the *Aspergillus* genus (Torres and Fraaije 2007). In 2004, *A*. *parasiticus* aflatoxin gene cluster was completely sequenced revealing the presence of at least one putative BVMO encoding ORF (Yu 2004a). The biosynthetic pathways for aflatoxins B_1_, G_1_ and B_2_, G_2_, starting from the early precursor nosrolinate anthrone, have been fully described (McGuire and Townsend 1993; Yu 2004b). However, not all the involved enzymes have been identified at the molecular/biochemical level. Moreover, the genome of *A*. *parasiticus* has not been completely sequenced, therefore the presence of other BVMO genes can not be ruled out. More recently, the cythochalasin gene cluster from *A*. *clavatus* NRRL 1 has been identified and engineered. It was demonstrated that within the multi-domain polyketide synthase-nonribosomal peptide synthetase complex (PKS–NRPS), responsible for the cytochalasin E and K synthesis, there might be a BVMO (CcsB) which displays a rare reaction on a vinyl carbonate moiety (Qiao 2011). The sequencing of the *A*. *fumigatus* Af293 genome was reported in 2005 (Nierman 2005). This allergenic pathogen is known to be a source of toxic compounds such as helvolic acid and fumagillin, which are probably the result of biosynthetic routes that involve BVMOs. Moreover, an early step catalyzed by a PKS complex leading to a lactone moiety, was identified in the biosynthesis of the meroterpenoid pyripyropene (Itoh 2010). In addition to the genetic and molecular evidence of BVMOs occurrence, there are many biotransformation reports where *A*. *fumigatus* catalyzes Baeyer-Villiger oxidations and enantioselective sulfoxidations (Jones 1994Mascotti 2012). These biochemical evidences support the presence of more than one BVMO in this species. So far, only one fungal BVMO has been produced as a recombinant enzyme: cycloalkanone monooxygenase (CAMO) from the ascomycete *Cylindrocarpon radicicola* (Leipold et al. 2011). All other available recombinant BVMOs belong to prokaryotic genomes.

Herein we describe the identification of a set of BVMOs from *Aspergillus fumigatus* Af293. In particular, we have cloned, expressed and biochemically characterized BVMO_Af1_, being the first *Aspergillus*-derived BVMO to be reported.

## Materials and methods

### Chemicals

All the reagents and chemicals were obtained from Across Organics and Sigma-Aldrich. Enzymes and oligonucleotides were purchased from Invitrogen, Sigma, NEB and Biodynamics. In-fusion™ 2.0 and Champion™ pET200 Directional TOPO® expression kits were purchased from Clonetech and Invitrogen, respectively.

### Data mining and searching for *Aspergillus fumigatus* BVMOs

The NCBI server was used for protein and DNA sequence retrieval by using BLASTP and TBLASTN under default parameters (http://blast.ncbi.nlm.nih.gov/Blast.cgi). By using PAMO and CHMO sequences (Bonsor 2006), *A*. *fumigatus* Af293 genomic, ESTs and protein databases were investigated for the presence of BVMO genes. The identified BVMO sequences were combined with the known bacterial BVMOs sequences and analyzed using CLUSTALW multiple alignment software (http://www.ebi.ac.uk/Tools/msa/clustalw2/). Further sequence analysis based on consensus motifs was made. Based on these sequence analyses, putative BVMO-encoding genes were identified.

### Cloning and gene expression

Genomic DNA from *A*. *fumigatus* Af293 (obtained from the FGSC) was purified as described before (Dellaporta 1983). The three selected BVMO-encoding genes; *Af1* (XM_742067), *Af2* (XM_741856) and *Af3* (XM_750181) were amplified by PCR using a proofreading polymerase (Phusion DNA polymerase, NEB). Full length PCR products were cloned into the pET200/D TOPO vector. Based on the sequence alignment and domain analyses, a truncated form of *Af1* (1614 bp), full length *Af2* and a truncated form of *Af3* (1632 bp) were also cloned into a pCRE2 vector by using the In-Fusion PCR Cloning kit (Clontech) (see online resource for details). This vector harbors a codon-optimized gene encoding a mutant of phosphite dehydrogenase (PTDH) with an N-terminal His-tag (Torres Pazmiño 2009b). For expression experiments, BL21(DE3) and TOP10 bacterial strains were transformed with pET200 and pCRE-based constructs, respectively. For expression optimization of pET200 constructs, cultures were grown at 37°C up to an OD_600_ of 0.5. IPTG was added at different final concentrations (1.0 and 0.5 mM) and cultures were grown for 6 h at four different temperatures (17, 24, 30 and 37°C). For TOP10 cells harboring pCRE2 constructs, expression was assessed using 24 deep square-well micro-titer plates with the sandwich cover system, shaking at 200 rpm. Cells were grown in the presence of different L-arabinose concentrations (0.002, 0.02, 0.2% *w*/*v*) at four different temperatures (17°C for 48 h, 24°C for 36 h, 30°C for 24 h and 37°C for 12 h). In both cases, cell extracts were obtained using DNase/lysozyme. Total extracts as well as clarified extracts were analyzed by SDS-PAGE upon blue staining (SimplyBlue™ SafeStain, Invitrogen) for detecting soluble expression of the enzyme.

### Purification of recombinant BVMO_Af1_

The His-tagged PTDH-BVMO_Af1_ was purified using the following procedure: TOP10 cells harboring the pCRE-*Af1* construct, from 500 mL culture (36 h, 24°C, 0.02% *w*/*v* arabinose) were harvested by centrifugation. Cells were resuspended in 20 mL of 50 mM Tris/HCl pH 8.0 buffer containing 150 mM KCl, 10% (*v*/*v*) glycerol, 0.5 mM DTT, 0.5 mM PMSF and 100 μM FAD. Cells were disrupted by sonication and subsequently centrifuged (15,000 g for 45 min at 4°C, JA-17 rotor, Beckman Coulter). Clarified cell extract was loaded on 2 mL of Ni^2+^ Sepharose HP (GE Healthcare) and incubated for 1 h at 4°C while gently mixing. Then, the column was washed with three column volumes of 50 mM Tris/HCl pH 8.0, followed by three column volumes of same buffer containing 5.0 mM imidazole. The protein was eluted in the same buffer containing 500 mM imidazole. To remove imidazole, yellow fractions containing FAD-bound protein were applied on an Econo-Pac 10DG desalting column (Bio-Rad) pre-equilibrated with buffer Tris/HCl pH 8.0. The desalted and aliquoted protein was stored at -80°C.

The integrity and purity of protein samples were evaluated by SDS-PAGE. To quantify the purified enzyme, the extinction coefficient of FAD bound to PTDH-BVMO_Af1_ was determined as described previously (Fraaije 2005). UV–Vis absorption spectra were collected on a Perkin-Elmer Lambda Bio40 spectrophotometer.

### Biocatalytic characterization

Substrate screening was made according to the protocol described by Riebel (2012). All substrates were tested at 2.5 mM. To analyze the enantioselectivity of PTDH-BVMO_Af1_, enzyme-mediated oxidations were performed using *rac*-bicyclo[3.2.0]hept-2-en-6-one and thioanisole as substrates. Reaction mixtures (1.0 mL) containing 50 mM Tris/HCl pH 8.0, 2.5 mM substrate, 50 μM NADPH, 10 mM phosphite and 1.0 μM enzyme were incubated for 4 h and/or 24 h at 25°C, 200 rpm. In the case of *rac*-bicyclo[3.2.0]hept-2-en-6-one, samples were taken at regular time-intervals. Reactions were quenched by addition of 1.0 mL ethyl acetate. The organic layer was dried over MgSO_4_ and subsequently analyzed by GC-FID as previously described (Kamerbeek 2003b).

### Enzyme activity

Enzyme activity was measured spectrophotometrically by monitoring the substrate-dependent decrease in NADPH at 340 nm (ϵ_340_ = 6.22 mM^-1^ cm^-1^). Reaction mixtures typically contained 50 mM Tris/HCl pH 8.0, 100 μM NADPH, 2.0 mM *rac*-bicyclo[3.2.0]hept-2-en-6-one and 0.05 to 0.35 μM of enzyme. All kinetic measurements were performed at 25°C using air-saturated buffers. To determine the coenzyme specificity, NAD(P)H concentrations ranging from 0.05 to 2.0 mM were tested. The observed rate constants were fitted with the Michaelis-Menten equation using SigmaPlot for Windows version 10.0.

Activity and thermal stability were measured spectrophotometrically by performing the reaction at different temperatures, ranging from 20 to 55°C. The melting point was determined by the previously described method Thermo-FAD (Forneris 2009). Activity and stability over pH were measured by performing the reaction at different pHs. The Britton & Robinson buffer (Thomas 1931Baldrian 2004) at a concentration of 50 mM was used in the pH range of 4.0-10.0.

The activity in the presence of 5% (*v*/*v*) of ethanol, methanol, *t*-butanol, dioxane and DMSO was tested. To check the enzyme stability, enzyme was pre-incubated for 1 h at room temperature in the presence of 5% (*v*/*v*) of the selected solvents, and remaining activity was measured spectrophotometrically. The same protocol was followed to test activity in other conditions such as the presence of salts, glycerol and different buffer types.

## Results

### Mining the *A. fumigatus* Af293 genome for BVMO-encoding genes

**Figure 1:**
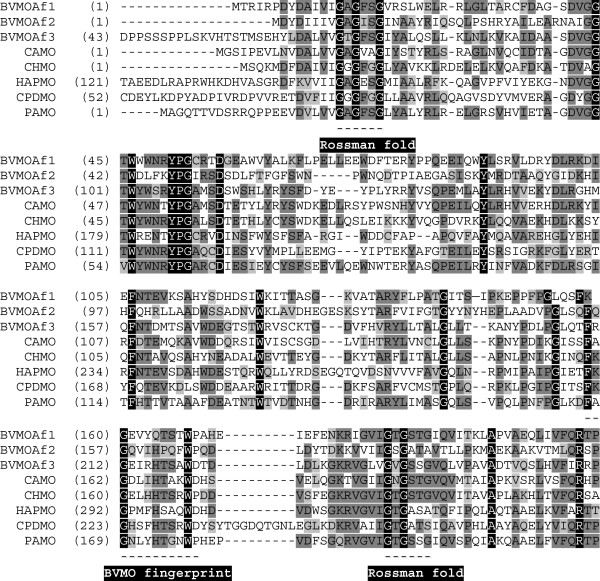
**Multiple sequence alignment of some BVMOs sequences.** Sequences are: BVMO_Af1_ (XP_747160), BVMO_Af2_ (XP_746949) and BVMO_Af3_ (XP_755274) from *A*. *fumigatus* Af293, PAMO (YP_289549) from *Thermobifida fusca*, CHMO (AAG10021) from *Acinetobacter* sp., HAPMO (AAK54073) from *Pseudomonas fluorescens*, CPDMO (BAE93346) from *Pseudomonas* sp. HI70 and CAMO (AET80001.1) from *Cylindrocarpon radicicola*. The two Rossmann folds (GxGxxG) and the BVMO fingerprint (FxGxxxHxxxWP/D) are highlighted.

By using the sequences of previously reported BVMOs (PAMO & CHMO) as probes, 13 predicted proteins from *A*. *fumigatus* with significant similarity (E-value < 1.10^-6^) were retrieved by BLASTP. No additional sequences were retrieved by TBLASTN on genomic and mRNA databases. The retrieved protein sequences were curated by confirming the presence of BVMO-typifying sequence hallmarks, namely two Rossmann folds GxGxx(G/A) flanking the FxGxxxHxxxW(P/D) motif (Fraaije 2002). Single amino acid substitutions were allowed in the latter motif (F/Y or H/Q). Nine predicted genes/proteins fulfilling these requirements were selected as putative BVMOs, XM_742067 (protein id XP_747160), XM_741856 (protein id XP_746949), XM_750181 (protein id XP_755274), XM_750991 (protein id XP_75684), XM_749026 (protein id XP_754119), XM_746162 (protein id XP_751255), XM_742681 (protein id XP_747774), XM_747111 (protein id XP_75224), XM_746209 (protein id XP_75132). These nine ORFs were aligned to genomic sequences to detect the presence of introns. Based on this, the three intronless nucleotide sequences XM_742067 (protein id XP_747160), XM_741856 (protein id XP_746949) and XM_750181 (protein id XP_755274) named *Af1*, *Af2* and *Af3* respectively, were chosen to be cloned and expressed (Figure 1). The enzymes encoded by the selected genes were named BVMO_Af1_, BVMO_Af2_, and BVMO_Af3_, according to their source strain, *A*. *fumigatus*.

The predicted BVMO_Af1_ contains 905 amino acids comprising an N-terminal BVMO domain (~540 residues) fused to a ~360 amino acids long putative polynucleotide synthase domain. For this reason, a truncated version of BVMO_Af1_ (1614 bp) was obtained by deleting 367 C-terminal residues. The two Rossmann fold motifs are conserved and confirm that the protein binds FAD and NADPH. Besides, in the BVMO fingerprint motif the conserved His residue is replaced by Gln.

BVMO_Af2_ is predicted to be a single, 486 amino acids-long, BVMO-domain. The full length encoding gene (1461 bp) was cloned in expression vectors. The BVMO fingerprint as well as the two Rossmann fold motifs are fully conserved in this protein.

The BVMO_Af3_ amino acid sequence presents an N-terminal extension of 58 residues compared to other BVMOs. In this case, a truncated fragment of 1632 bp was subcloned, in which the N-terminal extension was removed in order to prevent incorrect folding of the recombinant protein. This truncated enzyme comprised 543 amino acids and has canonical BVMO fingerprint and Rossmann fold motifs.

### Cloning and expression of recombinant *A. fumigatus* BVMOs

Two approaches were assessed. First, the three selected full-length sequences were cloned as 6×His fusion into a pET vector under the control of a T7 promoter. Small-scale expression conditions were tested for all these constructs. The pET-*Af1* and pET-*Af3* constructs yielded no soluble protein expression, as detected by SDS-PAGE. The pET-*Af2* construct allowed us to obtain soluble protein, although at a low expression level. In addition, the expressed BVMO_Af2_ did not show affinity towards the Ni^2+^ resin, preventing affinity chromatography purification. However, activity assays using crude extracts of *E*. *coli* cells expressing BVMO_Af2_ revealed that this enzyme is active (data not shown). In the case of the *Af1* gene, we hypothesize that the large C-terminal domain could be responsible for protein aggregation. Therefore, we tested the expression and solubility of the cloned BVMOs domains fused to an N-terminal His-tagged, cofactor regenerating protein; PTDH. Expression tests of these constructs revealed no detectable expression of BVMO_Af2_ or BVMO_Af3_, whereas BVMO_Af1_ yielded high expression of a soluble protein of the expected molecular weight. This result proves that fusion proteins may have a beneficial influence in the expression of non-soluble proteins, such it was the case of BVMO_Af1_, a fact that has been also reported for other BVMOs (Thapa 2008). Therefore, larger scale expression and purification were performed for this PTDH-BVMO fusion protein.

In the case of pCRE2-*Af2* construct the lack of expression may be due to the deletion of the N-terminal tail. For the gene *Af3*, functional expression was not achieved in any of the tested conditions indicating that a different expression system may be suitable in this case.

### Purification and spectral characterization of PTDH-BVMO_Af1_

**Figure 2:**
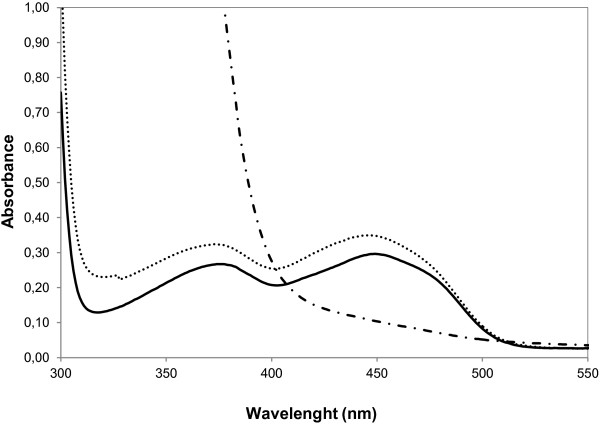
**Spectral characterization of BVMO**_**Af1**_**.** Visible spectra of native BVMO_Af1_ (solid line) and BVMO_Af1_ after unfolding by 1% SDS and incubation at 80°C (dotted line). Spectral changes observed upon reduction of BVMO_Af1_ by excess of NADPH (dashed line).

From a 2 L culture, 33 mg of His-tagged protein could be purified. The protein migrated as a single band on SDS-PAGE, corresponding to a mass of 100 kDa. This is in good agreement with the calculated mass for the PTDH-BVMO_Af1_ fusion protein (35 + 60.5 kDa). The yellow purified protein displayed two absorption maxima at 380 nm and 449 nm, as it is expected for a flavoprotein. The estimated extinction coefficient for the purified enzyme was determined as ϵ_449_ = 13.3 mM^-1^ cm^-1^. The addition of NADPH resulted in a complete reduction of the flavin evidenced by the spectral analysis, strongly suggesting that the recombinant protein is functional (Figure 2). The ratio of A_280_/A_449_ was found to be 14, indicating that the protein is mainly in its holo form.

### Catalytic properties

**Figure 3:**
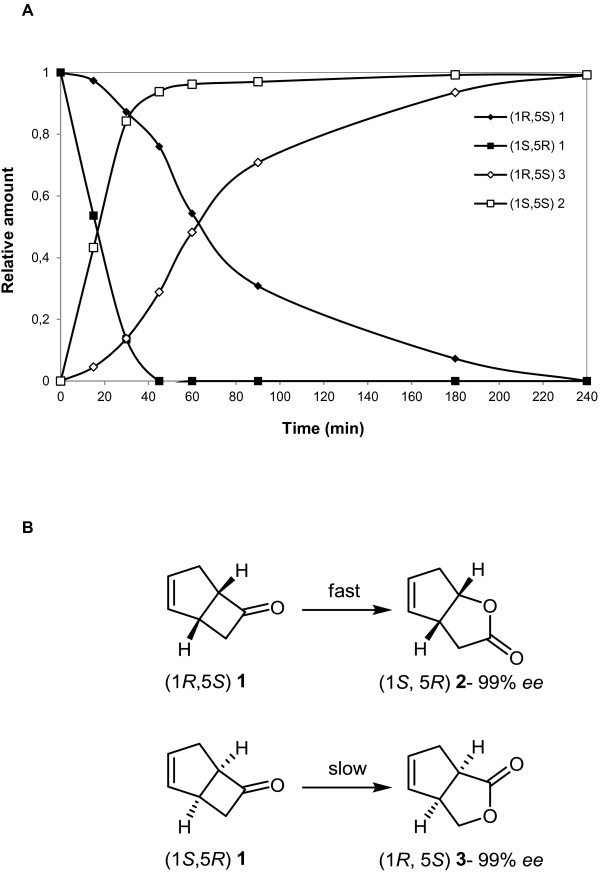
**Oxidation of *rac*-bicyclo[3.2.0]hept-2-en-6-one catalyzed by BVMO**_**Af1**_**.** (**A**) Time course BVMO_Af1_-catalyzed oxidation of *rac*-bicyclo[3.2.0]hept-2-en-6-one (1) to 2-oxabicyclo[3.3.0]oct-6-en-3-one (2) and 3-oxabicyclo[3.3.0]oct-6-en-2-one (3). (**B**) Structural formulas of bicyclo [3.2.0]hept-2-en-6-one isomers and lactones formed by BVMO_Af1_ activity.

In order to evaluate the biocatalytic potential of the recombinant enzyme, a set of 46 possible substrates was tested, including linear, cyclic, bicyclic and aromatic ketones, steroids, aromatic prochiral sulfides, diketones and bifunctional molecules. The assay revealed that four compounds namely *rac*-bicyclo[3.2.0]hept-2-en-6-one, benzyl ethyl sulfide, thioanisole and 3-phenylpentane-2,5-dione triggered significant NADPH consumption, which was evidenced by the phosphate production (Additional file 1: Table S2). In order to confirm the enzyme BVMO activity and to assess its enantioselectivity, reactions were carried out using the model substrate *rac*-bicyclo[3.2.0]hept-2-en-6-one since both enantioselectivity and regioselectivity, can be simultaneously tested (Mihovilovic 2005). In this reaction a kinetic resolution process takes place, the migration of the more-substituted carbon atom generates the “normal” lactone, while the migration of the less-substituted carbon atom forms the “abnormal” lactone (Renz and Meunier 1999). It was observed that BVMO_Af1_ converted the substrate in a highly selective fashion, successfully achieving the kinetic resolution of the substrate. The normal lactone (1*S*,5*R*)-2-oxabicyclo[3.3.0]oct-6-en-3-one (*ee* > 99%) was generated in a fast reaction, and full conversion was reached after 45 min. In contrast, the abnormal lactone (1*R*,5*S*)-3-oxabicyclo[3.3.0]oct-6-en-2-one (*ee* > 99%) was the product of a slow reaction and full conversion was achieved at 4 h (Figure 3). The ratio of the regioisomeric products was 1:1, with excellent optical purities of the formed lactones.

When thioanisole was tested as prochiral substrate, 88% conversion was achieved after 2 h. The (*S*)-sulfoxide was obtained with excellent enantioselectivity (*ee* > 99%). A similar optical purity was observed when the substrate was benzyl ethyl sulfide (Table 1). In both cases, sulfone formation was detected. In case of thioanisole, a small amount was produced after 2 h of conversion. On the other hand, in the case of benzyl ethyl sulfide the amount of sulfone was higher at shorter reaction times.

**Table 1:** **Sulfides conversion catalyzed by BVMOAf1**_**Af1**_

**Substrate**	**Time** (**min**)^**a**^	**Sulfoxide** (%)	**Sulfone** (%)	**ee** (%)
	180	88	8	>99 (*S*)
	60	56	37	>99 (*S*)
	180	25	75	>99 (*S*)

^a^ Reactions were monitored by sampling at regular time intervals. Full substrate conversion was observed at 180 min in both cases. For GC conditions, see online resource.

### Steady-state kinetic measurements

Steady-state kinetic parameters were determined for three of the substrates (Table 2). NADPH was found to be very well recognized by BVMO_Af1_ (K_*M*_ value < 5 μM). No significant activity was detected with NADH concentrations up to 1.0 mM, showing that this enzyme only accepts NADPH as hydride donor. BVMO_Af1_ highest apparent affinity was towards benzyl ethyl sulfide (K_*M*_ = 23.5 μM). The maximal rate of catalysis was in the same range for all the tested substrates, *k*_*cat*_ ≈ 0.5 s^-1^. This suggests that the conversion rates are independent of the type of substrate and hints to a rate-limiting step that does not involve the oxygenation reaction. Further kinetics analyses will reveal the rate limiting step during catalytic cycle.

**Table 2:** **Steady**-**state kinetic parameters of BVMO**_**Af1**_

***Substrate***	***K***_***M ***_**(μ** ***M*)**	***k***_***cat ***_**(*s***^**-** ***1***^**)**	***k***_***cat***_**/*K***_***M***_**(*s***^**-** ***1***^**/*mM***^**-** ***1***^**)**
	23.5 ± 0.1	0.47 ± 0.04	20
	118.8 ± 0.1	0.46 ± 0.02	4.1
	179.8 ± 0.1	0.36 ± 0.06	2.0

### Optimal conditions for activity & stability

**Figure 4:**
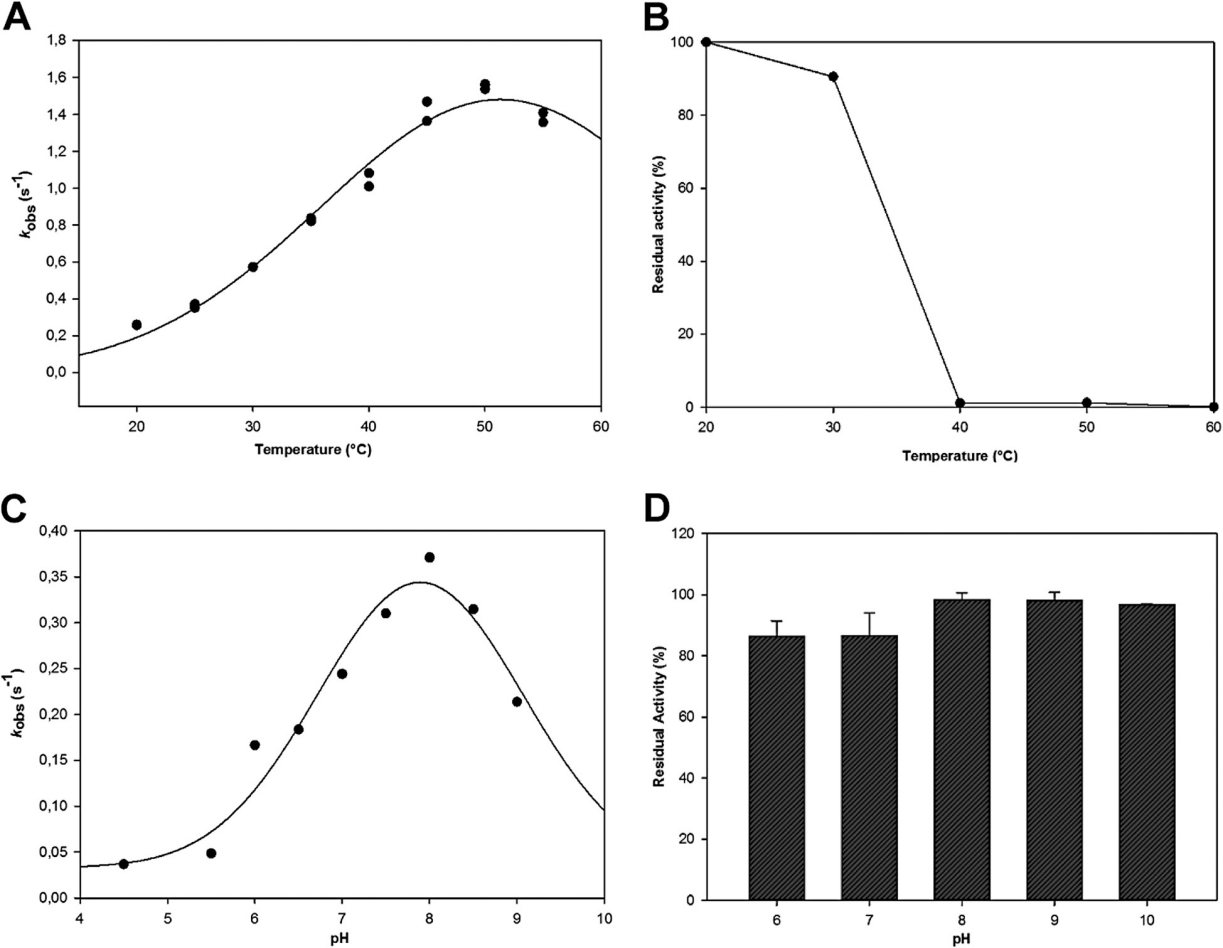
**Optimal conditions for activity.** (**A**) temperature-dependent activity profile, (**B**) temperature-dependent stability, for which the remaining activity was measured after 1.5 h incubation at the indicated temperatures, (**C**) pH-dependent activity profile, and (**D**) pH-dependent stability profile, by measuring the activity after 1 h incubation at different pH values. The error bars indicate standard deviations.

Optimal temperature was determined toward *rac*-bicyclo[3.2.0]hept-2-en-6-one. It was found that the enzyme exhibits its highest activity at 50°C (Figure 4-A). Based on this observation, the remaining activity was measured after 1.5 h of pre-incubation at different temperatures (20, 30, 35, 40, 50 and 60°C) and no significant loss of activity was detected up to 35°C (Figure 4-B). The melting temperature (T_m_) was 41.1°C and 43.3°C in Tris/HCl and phosphate buffers, respectively. The difference between both values could be attributed to the ionic nature of phosphate buffer, which may change the unfolding temperature, as it has been previously described for other enzymes (Cacace 1997). Alternatively, the inorganic phosphate may occupy binding pockets that are normally occupied by the phosphate moieties of NADP^+^ during catalysis, thereby stabilizing the enzyme.

The optimum pH for activity was determined to be 8.0, a typical value for Type I BVMOs (Figure 4-C). The effect of protein pre-incubation during 1 h at different pH values was also evaluated. Notably, the enzyme remained active at pHs ranged from 6.0 to 10.0 (Figure 4-D). Different buffers were tested but no significant changes were observed among them.

**Figure 5:**
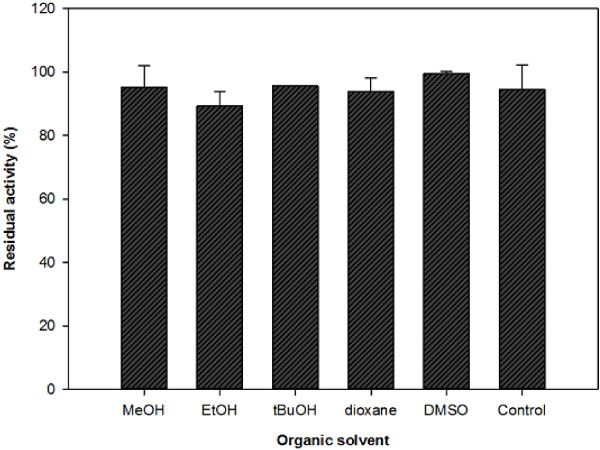
**BVMO**_**Af1 **_**stability in organic solvents.** Activity was measured after 1.0 h incubation in the presence of 5% (*v*/*v*) of organic solvents at room temperature. As control, no solvent was added to the buffer. The error bars indicate standard deviations.

The addition of organic solvents to enzyme-catalyzed procedures is usually used to improve the solubility of the substrate/product and/or to increase the reaction yield. Therefore, the activity of BVMO_Af1_ in the presence of ethanol, methanol, *t*-butanol, dioxane and DMSO (5% *v*/*v*) was evaluated. None of the solvents significantly influenced the enzyme activity. Based on this, enzyme stability towards these solvents was tested. Remarkably, the enzyme remained fully active after 1 h of pre-incubation with all the solvents, showing a very good tolerance to harsh reaction conditions (Figure 5).

## Discussion

In this study we have identified nine nucleotide sequences in the genome of *A*. *fumigatus* Af293 that contain all sequence hallmarks that, at the protein sequence level, are typical for Type I BVMOs. Interestingly, sequence identity among these predicted enzymes is rather low (ranging from 15% to 40%). Therefore, this fungus is a very interesting model to understand the genetic and molecular basis of the diversity of these enzymes within eukaryotic genomes, such as *Rhodococcus* is a model for prokaryotes (Balke 2012). Besides, from a biocatalytic point of view, this fact could explain the whole-cell activity of *A*. *fumigatus* toward a broad range of substrates (Bastos Borges 2009). Moreover, the poor stereoselectivity evidenced in some cases could be explained to the occurrence of different BVMOs with opposite selectivities (Mascotti 2012). To explore the biocatalytic potential of these fungal BVMOs, we have cloned three of these genes and succeeded in the effective expression of one of the targeted BVMOs, BVMO_Af1_. A thoroughly characterization of this first recombinant *Aspergillus* BVMO has been performed.

The BVMO_Af1_ primary sequence shows 37% identity with the only eukaryotic BVMO previously described, CAMO (Leipold 2012). It displays a higher degree of homology with bacterial BVMOs (40% for CHMO from *Acinetobacter* and 42% for PAMO from *Thermobifida fusca*). Interestingly, BVMO_Af1_ has an amino acid substitution (H173Q) in the consensus BVMO motif. This residue has been proposed to be involved in conformational changes during the catalytic cycle, acting as a linker between the FAD and NADPH binding sites (Malito 2004). The mutation of the central His residue in other BVMOs has dramatic effects, such as reduced catalytic efficiency in the case of CHMO (Cheesman 2003) or inactivation in the case of HAPMO (Fraaije 2002). However, wild type BVMO_Af1_ proved to be active and highly stereoselective. The observed mild mutation is also in line with the finding that some rhodococcal BVMOs do not have a fully conserved BVMO motif ( Riebel 2012).

Intriguingly, there are no shared structural features among the identified substrates for this new enzyme. Therefore, we have named it BVMO_Af1_, according to its source organism. The low number of identified substrates for BVMO_Af1_ can be related to the fact that the activity screening assay has been designed based on the reported substrates for prokaryotic BVMOs. Nevertheless, the observed enantioselectivity was excellent in the bicyclic ketone oxidation as well as in the oxidation of thioanisole and benzyl ethyl sulfide. As control, conversion of cyclohexanone and phenylacetone were evaluated. Although these two ketones are accepted by many of the known BVMOs, no conversion was detected with BVMO_Af1_ and the substrates were fully recovered. These results showed that the enzyme is highly chemo-, regio-, and stereoselective, proving its interesting biocatalytic potential. The exploration of different substrates is currently underway in our laboratory.

From the steady state kinetic analysis of BVMO_Af1_, it was found that the *k*_*cat*_ values are rather modest (0.5 s^-1^) and independent of the type of substrate. This fact led us to propose that a predetermined reaction rate is expected for any compound capable of entering into the active site and react with the peroxyflavin. This assumption suggests that a kinetic event before or after oxygenation is limiting the rate of catalysis. To determine if the catalytic mechanism of this eukaryotic protein resembles that of the well-studied bacterial BVMOs, more detailed kinetic studies are necessary. Also, a very strict dependence of NADPH as cofactor was determined. This is a typical feature of all reported BVMOs so far, except for MekA from *Pseudomonas veronii* which seems to accept either NADH or NADPH as hydride donors ( Völker et al. 2008).

By testing different parameters such as temperature, pH and the addition of organic solvents, it was demonstrated that BVMO_Af1_ is a robust biocatalyst. Remarkably, the pre-incubation of the enzyme either in a broad pH range or in organic solvents resulted in no loss of activity. For some other enzymes it has been shown that the presence of organic solvents in the reaction media, even without longer incubation times, dramatically reduces the activity. For instance, the prototype BVMO, cyclohexanone monooxygenase, loses dramatically its activity when it is incubated with 5% of solvents and its tolerance is very poor, since after 20 min incubation with 5% methanol the enzyme is completely inactivated ( Secundo 2011). These features, in combination with the biocatalytic properties of BVMO_Af1_, make this enzyme a promising biocatalyst.

BVMO_Af1_ has been extensively studied in this work, proving that this fungal enzyme has typical BVMO characteristics. However, there are some special features such as the substrate scope, the kinetic parameters and its high tolerance to harsh conditions that open new questions about eukaryotic BVMOs and encourage the work with other fungal enzymes belonging to this class.

## Competing interests

The authors declare that they have no competing interests.

## Supplementary Material

Additional file 1 Electronic Supplementary Material. Click here for file

## References

[B1] Baldrian P Purification and characterization of laccase from the white-rot fungus *Daedalea quercina* and decolorization of synthetic dyes by the enzyme Appl Microbiol Biotechnol 2004 3 560 563 10.1007/s00253-003-1434-0 14504838

[B2] Balke K Kadow M Mallin H Saß S Bornscheuer UT Discovery, application and protein engineering of Baeyer-Villiger monooxygenases for organic synthesis Org Biomol Chem 2012 3 6249 65 10.1039/c2ob25704a 22733152

[B3] Bastos Borges K De Souza BW Durán-Patrón R Tallarico Pupo M Sueli Bonato P González Collado I Stereoselective biotransformations using fungi as biocatalysts Tet Asymm 2009 3 385 397 10.1016/j.tetasy.2009.02.009

[B4] Bonsor D Butz SF Solomons J Grant S Fairlamb IJS Fogg MJ Grogan G Ligation independent cloning (LIC) as a rapid route to families of recombinant biocatalysts from sequenced prokaryotic genomes Org Biomol Chem 2006 3 1252 1260 10.1039/b517338h 16557313

[B5] Brondani PB De Gonzalo G Fraaije MW Andrade LH Selective oxidations of organoboron compounds catalyzed by Baeyer-Villiger Monooxygenases Adv Synth Catal 2011 3 2169 2173 10.1002/adsc.201100029

[B6] Cacace MG Landau EM Ramsden JJ The Hofmeister series: salt and solvent effects on interfacial phenomena Q Revs Biophys 1997 3 241 277 10.1017/S0033583597003363 9394422

[B7] Cheesman MJ Kneller MB Rettie AB Critical role of histidine residues in cyclohexanone monooxygenase expression, cofactor binding and catalysis Chem Biol Interact 2003 3 157 164 10.1016/S0009-2797(03)00105-4 14597129

[B8] Clouthier CM Kayser MK Increasing the enantioselectivity of cyclopentanone monooxygenase (CPMO): profile of new CPMO mutants Tet Asymm 2006 3 2649 2653 10.1016/j.tetasy.2006.10.001

[B9] Colonna S Gaggero N Carrea G Ottolina G Pasta P Zambianchi F First asymmetric epoxidation catalyzed by cyclohexanone monooxygenase Tet Lett 2002 3 1797 1799 10.1016/S0040-4039(02)00029-1

[B10] Dellaporta SL Wood J Hicks JB A plant DNA minipreparation: version II Plant Mol Biol Rep 1983 3 19 21 10.1007/BF02712670

[B11] Forneris F Orru R Bonivento D Chiarelli LR Mattevi A ThermoFAD, a Thermofluor®-adapted flavin ad hoc detection system for protein folding and ligand binding FEBS J 2009 3 2833 2840 10.1111/j.1742-4658.2009.07006.x 19459938

[B12] Fraaije MW Kamerbeek NA Van Berkel WJH Janssen DB Identification of a Baeyer-Villiger monooxygenase sequence motif FEBS Lett 2002 3 43 47 10.1016/S0014-5793(02)02623-6 11997015

[B13] Fraaije MW Wu J Heuts DPMH Van Hellemond EW Lutje Spelberg JH Janssen DB Discovery of a thermostable Baeyer–Villiger monooxygenase by genome mining Appl Microbiol Biotechnol 2005 3 393 400 10.1007/s00253-004-1749-5 15599520

[B14] Itoh T Tokunaga K Matsuda Y Fujii I Abe I Ebizuka Y Kushiro T Reconstitution of a fungal meroterpenoid biosynthesis reveals the involvement of a novel family of terpene cyclases Nature Chem 2010 3 858 864 10.1038/nchem.764 20861902

[B15] Jones KH Trudgill PW Hopper DJ 4-Ethylphenol metabolism by *Aspergillus fumigatus* Appl Environ Microbiol 1994 3 1978 1983 8031091 10.1128/aem.60.6.1978-1983.1994 PMC201590

[B16] Kamerbeek NM Janssen DB Van Berkel WJH Fraaije MW Baeyer-Villiger Monooxygenases, an emerging family of flavin-dependent biocatalysts Adv Synth Catal 2003 3 667 678 10.1002/adsc.200303014

[B17] Kamerbeek NM Olsthoorn AJJ Fraaije MW Janssen DB Substrate specificity and enantioselectivity of 4-hydroxyacetophenone monooxygenase Appl Environ Microbiol 2003 3 419 426 10.1128/AEM.69.1.419-426.2003 12514023 PMC152415

[B18] Leipold F Wardenga R Bornscheuer UW Cloning, expression and characterization of a eukaryotic cycloalkanone monooxygenase from *Cylindrocarpon radicicola* ATCC 11011 Appl Microbiol Biotechnol 2011 3 705 712 22075635 10.1007/s00253-011-3670-z

[B19] Leisch H Shi R Grosse S Morley K Bergeron H Cygler M Iwaki H Hasegawa Y Lau PCK Cloning, Baeyer-Villiger biooxidations, and structures of the camphor pathway 2-oxo-3-4,5,5-trimethylcyclopentenylacetyl-Coenzyme A monooxygenase of *Pseudomonas putida* ATCC 17453 Appl Environ Microb 2012 3 2200 2212 10.1128/AEM.07694-11 PMC3302634 22267661

[B20] Malito E Alfieri A Fraaije MW Mattevi A Crystal structure of a Baeyer-Villiger monooxygenase PNAS 2004 3 13157 13162 10.1073/pnas.0404538101 15328411 PMC516541

[B21] Mascotti ML Orden AA Bisogno FR De Gonzalo G Kurina-Sanz M *Aspergillus* genus as a source of new catalysts for sulfide oxidation J Mol Catal B: Enzym 2012 3 32 36

[B22] McGuire SM Townsend CA Demonstration of Baeyer-Villiger oxidation and the course of cyclization in bisfuran ring formation during aflatoxin B1 biosynthesis Bioorg Med Chem Lett 1993 3 653 656

[B23] Mihovilovic MD Rudroff F Grötzl B Kapitan P Snajdrova R Rydz J Mach R Family clustering of Baeyer-Villiger monooxygenases based on protein sequence and stereopreference Angew Chem Int 2005 3 3609 3613 10.1002/anie.200462964 15861400

[B24] Nierman WC Pain A Anderson MJ Wortman JR Kim HS Arroyo J Berriman M Abe K Archer DB Bermejo C Bennett J Bowyer P Chen D Collins M Coulsen R Davies R Dyer PS Farman M Fedorova N Feldblyum TV Fischer R Fosker N Fraser A García JL García MJ Goble A Goldman GH Gomi K Griffith-Jones S Gwilliam R Haas B Haas H Harris D Horiuchi H Huang J Humphray S Jiménez J Keller N Khouri H Kitamoto K Kobayashi T Konzack S Kulkarni R Kumagai T Lafton A Latgé JP Li W Lord A Lu C Majoros WH May GS Miller BL Mohamoud Y Molina M Monod M Mouyna I Mulligan S Murphy L O’Neil S Paulsen I Peñalva MA Pertea M Price C Pritchard BL Quail MA Rabbinowitsch E Rawlins N Rajandream MA Reichard U Renauld U Robson GD Rodriguez de Córdoba S Rodríguez-Peña JM Ronning CM Rutter S Salzberg SL Sanchez M Sánchez-Ferrero JM Saunders D Seeger K Squares R Squares S Takeuchi M Tekaia F Turner G Vazquez de Aldana GR Weidman J White O Woodward J Yu JH Fraser C Galagan JE Asai J Machida M Hall N Barrell B Denning DW Genomic sequence of the pathogenic and allergenic filamentous fungus *Aspergillus fumigatus* Nature 2005 3 22 29 10.1038/nature04332 16372009

[B25] Ottolina G De Gonzalo G Carrea G Theoretical studies of oxygen atom transfer from flavin to electron-rich substrates Theochem J Mol Struc 2005 3 175 181 10.1016/j.theochem.2005.05.023

[B26] Qiao K Chooi YH Tang Y Identification and engineering of the cytochalasin gene cluster from *Aspergillus clavatus* NRRL 1 Metab Eng 2011 3 723 732 10.1016/j.ymben.2011.09.008 21983160 PMC3254600

[B27] Renz M Meunier B 100 years of Baeyer-Villiger oxidations Eur J Org Chem 1999 737 750

[B28] Riebel A Dudek HM De Gonzalo G Stepniak P Rychlewski L Fraaije MW Expanding the set of rhodococcal Baeyer–Villiger monooxygenases by high-throughput cloning, expression and substrate screening Appl Microbiol Biotechnol 2012 3 1479 1489 10.1007/s00253-011-3823-0 22218769 PMC3427485

[B29] Secundo F Fialá S Fraaije MW De Gonzalo G Meli M Zambianchi F Ottolina G Effects of water miscible organic solvents on the activity and conformation of the baeyer–villiger monooxygenases from *thermobifida fusca* and *acinetobacter calcoaceticus*: a comparative study Biotechnol Bioeng 2011 3 491 499 10.1002/bit.22963 20939006

[B30] Sheng D Ballou DP Massey V Mechanistic studies of cyclohexanone monooxygenase: chemical properties of intermediates involved in catalysis Biochemistry 2001 3 11156 11167 10.1021/bi011153h 11551214

[B31] Szolkowy C Eltis LD Bruce NC Grogan G Insights into sequence-activity relationships amongst Baeyer-Villiger Monooxygenases as revealed by the intragenomic complement of enzymes from *Rhodococcus jostii* RHA1 ChemBioChem 2009 3 1208 1217 10.1002/cbic.200900011 19360806

[B32] Thapa A Shahnawaz M Karki P Raj Dahal G Golam Sharoar M Yub Shin S Sup Lee J Cho B Park I-S Purification of inclusion body-forming peptides and proteins in soluble form by fusion to *Escherichia coli* thermostable proteins Biotechniques 2008 3 787 796 10.2144/000112728 18476832

[B33] Thomas H Britton S Robinson RA Universal buffer solutions and the dissociation constant of veronal J Chem Soc 1931 1456 1462 10.1039/JR9310001456

[B34] Torres Pazmiño DE Baas BJ Janssen DB Fraaije MW Kinetic mechanism of phenylacetone monooxygenase from *Thermobifida fusca* Biochemistry 2009 3 4082 4093 10.1021/bi702296k 18321069

[B35] Torres Pazmiño DE Riebel A De Lange J Rudroff F Mihovilovic MD Fraaije MW Efficient biooxidations catalyzed by a new generation of self-sufficient Baeyer–Villiger Monooxygenases ChemBioChem 2009 3 2595 2598 10.1002/cbic.200900480 19795432

[B36] Torres P Fraaije MW Tomoko M Discovery, redesign and applications of Baeyer-Villiger monooxygenases Future directions in biocatalysis 2007 New York, USA: Elsevier BV 107 127

[B37] Van Beek HL De Gonzalo G Fraaije MW Blending Baeyer–Villiger monooxygenases: using a robust BVMO as a scaffold for creating chimeric enzymes with novel catalytic properties Chem Commun 2012 3 3288 3290 10.1039/c2cc17656d 22286124

[B38] Völker A Kirschner A Bornscheuer UT Altenbuchner J Functional expression, purification, and characterization of the recombinant Baeyer-Villiger monooxygenase MekA from *Pseudomonas veronii* MEK700 Appl Microbiol Biotechnol 2008 3 1251 1260 10.1007/s00253-007-1264-6 18034235

[B39] Yu J Bhatnagar D Cleveland TE Completed sequence of aflatoxin pathway gene cluster in *Aspergillus parasiticus* FEBS Lett 2004 3 126 130 10.1016/S0014-5793(04)00327-8 15094053

[B40] Yu J Chang PK Ehrlich KC Cary JW Bhatnagar D Cleveland TW Payne GA Linz JE Woloshuk CP Bennett JW Clustered pathway genes in aflatoxin biosynthesis Appl Environ Microbiol 2004 3 1253 1262 10.1128/AEM.70.3.1253-1262.2004 15006741 PMC368384

[B41] Zhang ZG Roiban GD Acevedo JP Polyak I Reetz MT A new type of stereoselectivity in Baeyer–Villiger reactions: access to E- and Z-olefins Adv Synth Catal 2013 3 99 106 10.1002/adsc.201200759

