# Erratum to: Cloning, overexpression and biocatalytic exploration of a novel Baeyer-Villiger monooxygenase from *Aspergillus fumigatus* Af293

**DOI:** 10.1186/s13568-014-0062-7

**Published:** 2014-09-20

**Authors:** Maria Laura Mascotti, Maximiliano Juri Ayub, Hanna Dudek, Marcela Kurina Sanz, Marco W Fraaije

**Affiliations:** INTEQUI-CONICET, Facultad de Química Bioquímica y Farmacia, Universidad Nacional de San Luis, San Luis, CP 5700 Argentina; IMIBIO-CONICET, Facultad de Química Bioquímica y Farmacia, Universidad Nacional de San Luis, San Luis, CP 5700 Argentina; Laboratory of Biochemistry, Groningen Biomolecular Sciences and Biotechnology Institute, University of Groningen, Nijenborgh 4, Groningen, 9747 AG The Netherlands

## Correction

In the legend of Figure 2 of the original version of this article (Mascotti et al. [2013]) the caption read as follows:

**Figure 2**. Spectral characterization of BVMO_Af1_. Visible spectra of native BVMO_Af1_ (solid line) and BVMO_Af1_ after unfolding by 1% SDS and incubation at 80°C (dotted line). Spectral changes observed upon reduction of BVMO_Af1_ by excess of NADPH (dashed line).

The listing of ‘(solid line)’ and ‘(dotted line)’ are incorrectly placed in the text and in fact should be located in reverse. The caption should correctly read as follows:

**Figure 2.** Spectral characterization of BVMO_Af1_. Visible spectra of native BVMO_Af1_ (dotted line) and BVMO_Af1_ after unfolding by 1% SDS and incubation at 80°C (solid line). Spectral changes observed upon reduction of BVMO_Af1_ by excess of NADPH (dashed line).
