# Supplementary material for: Cloning, overexpression and biocatalytic exploration of a novel Baeyer-Villiger monooxygenase from Aspergillus fumigatus Af293
Source: AMB Express. 2013 Jun 14;3:33. doi: 10.1186/2191-0855-3-33 (PMC3762062; doi:10.1186/2191-0855-3-33)
Supplement: Additional file 1 — Electronic Supplementary Material. [file 2191-0855-3-33-S1.pdf]

CLONING, OVEREXPRESSION AND BIOCATALYTIC EXPLORATION OF A NOVEL BAEYER-VILLIGER MONOOXYGENASE FROM *ASPERGILLUS FUMIGATUS* AF293

Maria Laura Mascotti[1], Maximiliano Juri Ayub[2], Hanna Dudek[3], Marcela Kurina Sanz*[1], Marco W. Fraaije**[3]

[1] INTEQUI-CONICET, Facultad de Química Bioquímica y Farmacia, Universidad Nacional de San Luis, CP 5700, San Luis, Argentina

[2] IMIBIO-CONICET, Facultad de Química Bioquímica y Farmacia, Universidad Nacional de San Luis, CP 5700, San Luis, Argentina

[3] Laboratory of Biochemistry, Groningen Biomolecular Sciences and Biotechnology Institute, University of Groningen, Nijenborgh 4, 9747 AG, Groningen, The Netherlands.

Corresponding authors:

*Marcela Kurina-Sanz

E-mail: marcelakurina@gmail.com; Tel: +54-0266-4439909

**Marco W. Fraaije

E-mail: m.w.fraaije@rug.nl; Tel: +31 50-363-4345

Electronic Supplementary Material

Contents

1. PCR protocols: Primers & Vectors
2. Expression conditions
3. Expression of $BVMO_{Af1}$
4. Substrates screening
5. GC analyses
6. *Aspergillus fumigatus* Af293 BVMO encoding genes

1. PCR protocols: Primers & Vectors

Table S1

Cloned genes, expression vectors and primers used for genomic DNA amplification. Restriction sites are underlined

Gene	***Size***	***Vector***	***Oligonucleotides 5'→3'***
Af1	2718	pET200	Fw: CACCATGACCAGAATACGTCCAGAC<br>Rev_EcoRI: CGGAATTCTCAATGCCCATTAGTAGTAACGG
Af1 truncated	1614	pCRE2	Fw: ACTCGAGATCTGCAGCTGGTATGACCAGAATACGTCCAG<br>Rev: GTTCGGGCCCAAGCTTTAACGTGTAAAGCTCATA
Af2	1461	pET200	Fw_Nde I: CACCCATATGGATTACGATATTATCATTGTTGG<br>Rev_Hind III: GCGAAGCTTCTATTGCTGCTTCTTCCAGCC
		pCRE2	Fw: ACTCGAGATCTGCAGCTGGTATGGATTACGATATTATCA<br>Rev: GTTCGGGCCCAAGCTTTATTGCTGCTTCTTCCAG
Af3	1806	pET200	Fw_Nde I: CACCCATATGCGTTGCATACCCTGCC<br>Rev_Eco RI: GCGGAATTCTTATAGAAGCGGCCGCGGC
Af3 truncated	1632	pCRE2	Fw: ACTCGAGATCTGCAGCTGGTATGTCAGAACACTACCTCG<br>Rev: GTTCGGGCCCAAGCTTTATAGAAGCGGCCGCGGC

2. Expression conditions

Table S2

All the expression conditions assayed are listed below

Vector	***Cells***	***Growing T***[a]	***Inductor***	***Induction T***	***Induction time***
pET-*Af1*<br>pET-*Af2*<br>pET-*Af3*	BL21	37 °C<br>(OD_{600}=0.5)	IPTG<br>0.5 mM	17°C<br>24°C<br>30°C<br>37°C	6 h
pET-*Af1*<br>pET-*Af2*<br>pET-*Af3*	BL21	37 °C<br>(OD_{600}=0.5)	IPTG<br>1 mM	17°C<br>24°C<br>30°C<br>37°C	6 h
pCRE-*Af1*<br>pCRE-*Af2*<br>pCRE-*Af3*	TOP 10	-	Arabinose<br>0.002% *w/v*	17°C	48 h
				24°C	36 h
				30°C	24 h
				37°C	12 h
pCRE-*Af1*<br>pCRE-*Af2*<br>pCRE-*Af3*	TOP 10	-	Arabinose<br>0.02% *w/v*	17°C	48 h
				24°C	36 h
				30°C	24 h
				37°C	12 h
pCRE-*Af1*<br>pCRE-*Af2*<br>pCRE-*Af3*	TOP 10	-	Arabinose<br>0.2% *w/v*	17°C	48 h
				24°C	36 h
				30°C	24 h
				37°C	12 h

[a] For pCRE2 based constructs, no pre-culture is needed, growing is directly in the presence of inducer

3. Expression of $BVMO_{Af1}$

Figure S1

SDS-PAGE analyses for $BVMO_{Af1}$ purification fractions. *lane 1:* crude extract, lane *2:* flow through, lanes *3, 4,* and *5:* sequential washes with imidazole increasing concentrations, *lane 6:* yellow, active fraction. The arrow refers to purified $BVMO_{Af1}$

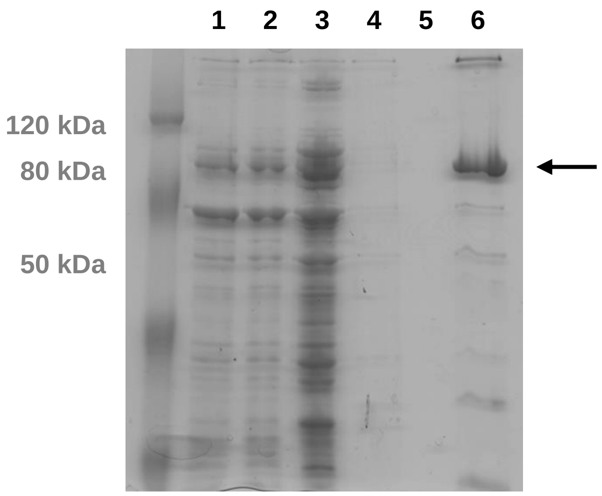

4. Substrates screening

Table S3

$BVMO_{Af1}$ substrates profile

Entry	*Substrate*	*$BVMO_{Af1}$* [a]
1	2-propanone	
2	2-butanone	
3	3-buten-2-one	
4	2-octanone	
5	3-octanone	
6	4-octanone	
7	2-decanone	
8	butyl levulinate	
9	3-methyl-2,4-pentanedione	
10	cyclobutanone	
11	cyclopentanone	
12	cyclohexanone	
13	cyclopentadecanone	
14	2-oxocyclohexanecarbonitrile	++
15	4-methyl cyclohexanone	
16	2-propyl cyclohexanone	
17	dehydrocarvone	
18	cyclopropyl methyl ketone	
19	norcamphor	
20	bicyclo[3.2.0]hept-2-en-6-one	+++
21	progesterone	
22	androstenedione	
23	4-dimethylamino benzaldehyde	
24	nicotine	
25	thioanisole	+

26	benzyl ethyl sulfide	+++
27	benzyl phenyl sulfide	+
28	ethionamide	+
29	diphenylmethylthioacetamide	
30	thiacetazone	
31	indole	
32	3-acetyl indole	
33	5-methyl furfural	
34	benzaldehyde	
35	acetophenone	
36	4-hydroxyacetophenone	
37	2,6-dihydroxy acetophenone	
38	3-phenylpentane-2,4-dione	+++
39	phenylacetone	
40	4-(4-hydroxyphenyl)-2-butanone	
41	2-phenyl cyclohexanone	
42	benzoin	
43	phenindione	
44	2-indanone	
45	1-indanone	

[a] Activity was measured employing a colorimetric (phosphate- based detection) screening assay, previously reported for BVMOs substrate screening (Riebel et al. 2012). The activity is indicated as +, ++ or +++ representing 1.2-, 2- or 5-fold phosphate formation (substrate conversion) respectively, when comparing with the blanks

5. GC analyses

The following columns were used for the determination of conversions and enantiomeric excesses: Column A: Alltech GT-A (30 m x 0.25 mm x 0.25 µm, 12.2 psi N_2); column B: Hewlett Packard HP-1 (30m x 0.32 mm x 0.25µm, 12.2 psi N_2) and C: Chirasil Dex CB (30 m x 0.25 mm x 0.25 µm, 12 psi N_2). For all the analyses, the injector temperature was 200ºC and the FID temperature was 250ºC

Table S4

GC employed conditions and retention times (t_R) of substrates and products

compound	***program***[a]	***column***	***t_R (min) substrates***	***t_R (min) products***
**1**	130°C isotherm	C	9 (1*S*,5*R*)<br>9.2 (1*R*,5*S*)	16.5 Abnormal (1*R*,5*S*)<br>16.9 Normal (1*R*,5*S*)<br>17.2 Abnormal (1*S*,5*R*)<br>17.4 Normal (1*S*,5*R*)
**2**	70/0/5/200/0	B	9.63	13.4 sulfoxide<br>14.2 sulfone
	100/0/10/160/8	A	6.9	13.5 (*R*)<br>14.9 (*S*)
**3**	70/5/5/200/5	B	12.5	16.5 sulfoxide<br>16.9 sulfone
	40/0/10/160/8	A	8.8	14.4 (*R*)<br>15.5 (*S*)

[a] Program: initial T (ºC)/ time (min)/ slope (ºC/min)/T (ºC)/ time (min)/ slope (ºC/min)/T (ºC)/ time (min).

Bicyclo[3.2.0]6hepten-2-one (**1**), thioanisol (**2**), benzyl ethyl sulfide (**3**)

6. *Aspergillus fumigatus* Af293 BVMO encoding genes

Figure S2

Multiple sequence alignment of *A. fumigatu*s Af293 BVMOs sequences. Sequences are: $BVMO_{Af1}$ (XP_747160), $BVMO_{Af2}$ (XP_746949), $BVMO_{Af3}$ (XP_755274), XP_751302, XP_747774, XP_754119, XP_752204, XP_756084 from *A. fumigatus* Af293, PAMO (YP_289549) from *Thermobifida fusca*, CHMO (AAG10021) from *Acinetobacter sp*., and CAMO (AET80001.1) from *Cylindrocarpon radicicola*. The two Rossmann folds (GxGxxG) and the BVMO fingerprint (FxGxxxHxxxWP/D) are in bolds

```
                         61                                                        120
BVMOAf1_Af293    (5)  RPDYDAIVIGAGFSGVRSLWELR----RLGLTARCFDAGSDVGGTWWWNRYPGCRTDGEA
BVMOAf2_Af293    (1)  -MDYDIIIVGAGISGINAAYRIQS--QLPSHRYAILEARNAIGGTWDLFKYPGIRSDSDL
BVMOAf3_Af293   (61)  EHYLDALVVGTGFSGIYALQSLL----KLNLKVKAIDAASDVGGTWYWSRYPGAMSDSWS
         PAMO   (14)  PEEVDVLVVGAGFSGLYALYRLR----ELGRSVHVIETAGDVGGVWYWNRYPGARCDIES
         CAMO    (7)  VLNVDALVVGAGVAGIYSTYRLS----RAGLNVQCIDTAGDVGGTWYWNTYPGAMSDTET
         CHMO    (4)  KMDFDAIVIGGGFGGLYAVKKLRD---ELELKVQAFDKATDVAGTWYWNRYPGALSDTET
    XP_751302   (51)  LPKKRVAVIGAGLTGVSSAAHCIG----HGFDVQLFEARP---------KDKGLGGIWSV
    XP_747774   (32)  ARPLRVVIIGSGISGIIASIRFRQR--IPNVDLCVYEKNEDIGGTWLENRYPGCACDIPA
    XP_754119   (54)  VRQIKVGVIGAGLSGITAGVLLPAK--LPGLDLRIYDKNADVGGTWFENTYPGVRCDIPA
    XP_751255   (29)  PRKLRVVCIGAGFSGLILAYKLKHERPIDFVDYTIYEKNPEVGGTWYENVYPGVGCDIPA
    XP_752204   (19)  HTYSPVLVIGAGVSGIATGCRLKEA--LGFDQFRIFERQSGIGGTWWINRYPGAACDVPA
    XP_756084   (37)  FTRATAVIIGAGISGICTAIDLIKR—-SKCHNFIILEKSSGVGGTWNDNRYPGSCCDVWS
                                GXGXXG
                         121                                                       180
BVMOAf1_Af293   (61)  WVYALKFLPELLEEWDFTERYPPQEEIQWYLSRVLDRYDLRKDIEFNTEVKSAHYSDHDS
BVMOAf2_Af293   (58)  FTFGFSWNPWN-----QDTPIAEGASISKYMRDTAAQYGIDKHIHFQHRLLAADWSSADN
BVMOAf3_Af293  (117)  HLYRYSFDYE----YPLYRRYVSQPEMLAYLRHVVEKYDLRGHMQFNTDMTSAVWDEGTS
         PAMO   (70)  IEYCYSFSEEVLQEWNWTERYASQPEILRYINFVADKFDLRSGITFHTTVTAAAFDEATN
         CAMO   (63)  YLYRYSWDKEDLRSYPWSNHYVYQPEILQYLRHVVERHDLRKYIRFDTEMQKAVWDDQRS
         CHMO   (61)  HLYCYSWDKELLQSLEIKKKYVQGPDVRKYLQQVAEKHDLKKSYQFNTAVQSAHYNEADA
    XP_751302   (98)  HSIMYRFHPS----VKYTTAYPSQQEIRDQIIDVWKRYGLQKRTAFDTPVTSVKQTK-DG
    XP_747774   (90)  HTYQATFEPN----KEWSTFYAAAPEIHAYWKRVAEKYGCMKYIKLKQAVVEAVWDDSKS
    XP_754119  (112)  HVYQSGFEPN----TQWTEEFAQGHEIREYWQRVARKYDVYKYLRPRQKVQKVVWRPEEA
    XP_751255   (89)  HSYVFPFEPN----PNWSKFYVSGPEIQDYIVKTTDKYGLRDKITFNTKLLQVAWDEGDG
    XP_752204   (77)  LLYSFSFAPK----KDWTTLHPPGPEIVQYLADVCEKYQIVDKIQLNTAVKEMRWLEDAE
    XP_756084   (95)  SLYSYSFEQK----ADWTREYPGQEEILDYLIGVAQKYGLYRYIRFNSAVEEARWDEADL

                         241                                                       300
BVMOAf1_Af293  (151)  PFPGLQSFKGEVYQTSTWPAHEIEFENKRIGVIGTGSTGIQVITKLAP---VAEQLIVFQ
BVMOAf2_Af293  (148)  DVPGLSQFQGQVIHPQFWPQD-LDYTDKKVVIIGSGATAVTLLPKMAE---KAAKVTMLQ
BVMOAf3_Af293  (203)  DLPGLQTFRGEIRHTSAWDTD-LDLKGKRVGLVGVGSSGVQLVPAVAD---TVQSLHVFI
         PAMO  (160)  NFPGLKDFAGNLYHTGNWPHEPVDFSGQRVGVIGTGSSGIQVSPQIAK---QAAELFVFQ
         CAMO  (153)  DIKGISSFAGDLIHTAKWDHS-VELQGKTVGIIGNGSTGVQVMTAIAP---KVSRLVSFQ
         CHMO  (151)  NIKGINQFKGELHHTSRWPDD-VSFEGKRVGVIGTGSTGVQVITAVAP---LAKHLTVFQ
    XP_751302  (183)  PLPDQGRFKGKIFHSSELDGK--DVEGKKVLIIGGGASAIEALEFAVKS--KARAIDVLS
    XP_747774  (181)  DIPGLHDFKGKLQHSARWDES-YDYTGKRAAVIGNGSSGIQIVPGMLP---KVAHIDHYI
    XP_754119  (203)  DYEGINDYQGDLFHSSNWNHN-VELKGKRVALIGNGASGLQVLPSIQP---IAAHVDHYA
    XP_751255  (178)  DVEGLNLFQGKLLHSARWDPD-YDWTGKGIAVIGNGSSALQIVPELQP---KAAKIVNYI
    XP_752204  (193)  TVPGIETFEGEVVHTARWKSD-LDLCGKDVVIIGSGCSATQVVSELTKPEYKIRSITQLM
    XP_756084  (191)  DIPGLDDYKGKLMHSARWDWS-FDWTGKRVAVIGNGATAAQIIPEIAK---TAAHVSVYQ
                              FXGXXXHXXXW(P/D)          GXGXX(G/A)
```
